# Impact of Water Purity and Oxygen Content in Gas Phase on Effectiveness of Surface Cleaning with Microbubbles

**DOI:** 10.3390/ma17246046

**Published:** 2024-12-10

**Authors:** Karol Ulatowski, Patryk Szczygielski, Paweł Sobieszuk

**Affiliations:** Department of Biotechnology and Bioprocess Engineering, Faculty of Chemical and Process Engineering, Warsaw University of Technology, Waryńskiego 1, 00-645 Warsaw, Poland

**Keywords:** microbubbles, cleaning, microbubble dispersion, surfactant removal, flotation

## Abstract

Cleaning of surfaces without complex cleaning agents is an important subject, especially in food, pharmaceutical, and biomedical applications. The subject of microbubble and nanobubble cleaning is considered one of the most promising ways to intensify this process. In this work, we check whether and how the purity of water used for microbubble generation, as well as the gas used, affects the effectiveness of cleaning stainless-steel surfaces. Surfaces contaminated with Pluronic L-121 solution were cleaned by water of three purities: ultrapure water (<0.05 μS/cm), water after reversed osmosis (~6.0 μS/cm), and tap water (~0.8 mS/cm). Similarly, three different gases were supplied to the generation setup for microbubble generation: air, oxygen, and nitrogen. Stainless steel plates were immersed in water during microbubble generation and cleaned for a given time. FTIR (Fourier Transform Infrared Spectroscopy) and contact angle analysis were employed for the analysis of surfaces. The results of cleaning were repeatable between plates and showed different cleaning effects depending on both the purity of water (concentration of ions) and gas composition. We have proposed different mechanisms that are dominant with respect to specific combinations of ion concentration and oxygen content in gas, which are directly connected to the microbubble stability and reactivity of gas.

## 1. Introduction

Cleaning of surfaces with nanobubbles and microbubbles is an interesting topic for both academia and the industry. Wu et al. (2006, 2007) have shown that bovine serum albumin (BSA) is effectively removed from highly oriented pyrolytic graphite by nanobubble-enriched water [1,2]. Similarly, Chen et al. (2009) have shown that BSA is also removed from stainless steel [3]. Later, Zhu et al. (2016) have shown that nanobubbles can not only detach contaminants from solid surfaces but also prevent those surfaces from being recontaminated [4]. Such a statement is connected mostly to the existence of surface nanobubbles, which are the objects of interest for multiple research groups worldwide (see, e.g., [5,6,7,8,9,10,11,12]). Yasui et al. (2023) have shown that the existence of surface nanobubbles on the solid surface changes the shape of droplets put onto it [13]. Besides BSA, which is the model contaminant of solid surfaces, there are not many data concerning submerged surface cleaning with nanobubbles. One of our previous works summarized our findings concerning the cleaning of oil-contaminated surfaces, where vegetable oil and UV-protective oils on glass and stainless steel were investigated [14]. We have shown that the longer the exposition to the nanobubble stream, the better the cleaning effect.

The subject, which is directly connected to submerged surface cleaning, is flotation. Flotation, which is needed to carry away the contamination from cleaned surfaces, can be realized using nanobubbles or microbubbles, which was proven by multiple research groups (see, e.g., [15,16,17,18,19,20,21,22,23,24,25,26]). As those fine bubbles are diminutive in size, they do not rise to the free surface of the liquid with significant velocity; more precisely, their rising velocity is even smaller than the velocity of Brownian motion and is, therefore, negligible. That means that bulk fine bubbles, which are dispersed in liquid, will be suspended in liquid until they meet some contamination. Many researchers have shown that fine bubbles, namely microbubbles and nanobubbles, can adhere to contamination and assist in flotation [16,27,28,29]. As they adhere in large numbers to the contaminant particles, they either allow for the adhesion of multiple larger bubbles to the coarse particles or the adhesion of fine particles to the surface of larger bubbles, depending on the size of the contaminants. Both mechanisms can potentially be either useful or detrimental when it comes to both cleaning and adhesion intensification or prevention. Additionally, it is a known fact that nanobubbles adhere to mineral surfaces, causing them to increase their hydrophobicity [30,31]. The work of Gomez-Florez et al. (2024) presents the modeling of bubble flotation and includes the hydrophobicity-enhancing effect of bubbles, which adhered to materials from spent lithium-ion battery electrodes. Such an effect varies depending on the size of the bubbles [30]. Kyzas et al. (2019) investigated the adhesion of lead cations onto the activated carbon and showed that nanobubbles increase the maximum solute uptake of carbon dioxide by about 2.5% [26]. The authors claim that this effect is connected to the adsorption of lead cations on negatively charged nanobubbles, therefore forming the positively charged surface. Such ions can interact with the negatively charged surface of activated carbon, causing their adhesion. Bubbles serve as carriers of cations, which can transfer cations to the porous surface of activated carbon. Ren et al. (2023) [25] have investigated the interactions between nanobubbles and cassiterite particles during flotation. They showed that nanobubbles enhance the effectiveness of the flotation process, which used classical flotation, by about 10–20%. What is interesting is that non-aqueous solutions have started to gain attention as the liquid media for fine bubble dispersion generation [32,33,34,35].

Other works show the usefulness of micro- and nanobubbles in the defouling and cleaning of membranes [36,37]. The addition of fine bubbles to the inlet flow causes slower contamination of membranes, less clogging, and reduction in viscosity of such streams, thus increasing the effectiveness of membrane separation. Additionally, such bubbles can also reverse fouling effects, causing a higher recovery of flux after cleaning.

The above references clearly show that nanobubbles and microbubbles can be used for cleaning surfaces. However, because nanobubble generation is often more expensive than microbubble generation, an investigation of microbubble cleaning and an understanding of its mechanism are still viable. As microbubble size can be controlled by changing the parameters of the experimental setup or process parameters [38,39,40,41,42], obtaining microbubbles tailored to the specific application is possible. Li et al. (2022) have investigated the cleaning of metal surfaces with air microbubbles obtained by a cavitation-based generator [43]. Surfaces were contaminated with machine oil and then degreased using microbubbles. After 15 min of cleaning, over 65% of the contamination was removed. The interesting fact is that the temperature of the dispersion mattered; for initial cleaning (5 min), the cleaning efficiency rose with temperature, while the overall effectiveness (after 15 min) was best for lower temperatures. Additionally, the authors observed intensive rusting of surfaces in higher temperatures. Therefore, keeping the temperature low seems to be appropriate for microbubble cleaning. As the literature suggests, micro-nanobubbles offer the option of environmentally friendly cleaning of surfaces [44,45], but the mechanisms of such cleaning have yet to be deeply investigated.

Efficient cleaning without detergents is especially important in the case of medical and biological studies and applications. Therefore, the contamination chosen was the surfactant commonly used in biological laboratories: Pluronic L121. In this work, we wish to tackle the subject of cleaning this contamination off the stainless-steel surfaces using microbubbles of various gases.

We feel that a significantly new element in the presented results is the use of water with distinct levels of purification and, more specifically, different contents of different ions. It is already clear that there is an influence of both ions [10,46,47] and hydrophobic impurities [13,48,49] on the stability and behavior of fine bubbles and surface tension of gas dispersion. Therefore, we expected that the purity of the water would also affect the effect of cleaning the surface with microbubble dispersions, especially in the case where the cleaned substance is an amphiphilic surfactant.

## 2. Results and Discussion

By design, the population of bubbles themselves should not change significantly during the process of cleaning for a given pair of water purity and gas. We performed the experiments in a large volume of microbubble dispersion, compared to the volume of surfactant added to the surface of the plates (below 1 mL for one plate compared to the 50 L of water in the system), to ensure the minimalization of the effect of surfactant on the microbubble generation process. Additionally, as the generation was ongoing during the cleaning process, the bubbles themselves were renewed, and as the generation parameters remained constant, the population of bubbles did not change as a result. For that reason, our system needs to have the appropriate mechanical strength. Therefore, we have decided to use a steel tank, which is opaque, and the optical methods of bubble size measurement are also not viable without bypassing the flow, which would affect the generation of microbubbles. Therefore, we are forced to rely on the manufacturer’s claims concerning the microbubble size distribution, i.e. the diameter of generated bubbles is in range between 8 and 15 μm.

Table 1 summarizes the data obtained from the analysis of the contact angle. From each frame, the right angle and left angle were measured, which were then averaged to obtain the median angle. As the droplets were mostly symmetrical, the values of both right and left angles were similar. Therefore, the median angle was considered.

As one can see in Table 1, values of contact angle measured for clean stainless-steel plates were equal to 82.01°±1.39°, while the contaminated surfaces exhibited higher wettability with water, i.e., the contact angle of 53.15°±3.01°. To check whether the cleaning would occur without microbubble flow, we also immersed the plates in water of all three purities for 30 min. The values of contact angle for such plates were equal to 50.28°±1.86°, 54.83°±1.37°, and 58.75°±3.00°, for UPW, ROW and TW, respectively. As the differences between the contaminated sample and samples immersed in water were minimal, we state that no significant cleaning occurred. Therefore, we showed that a different approach is needed and started the tests with microbubble dispersions.

**Figure 1:**
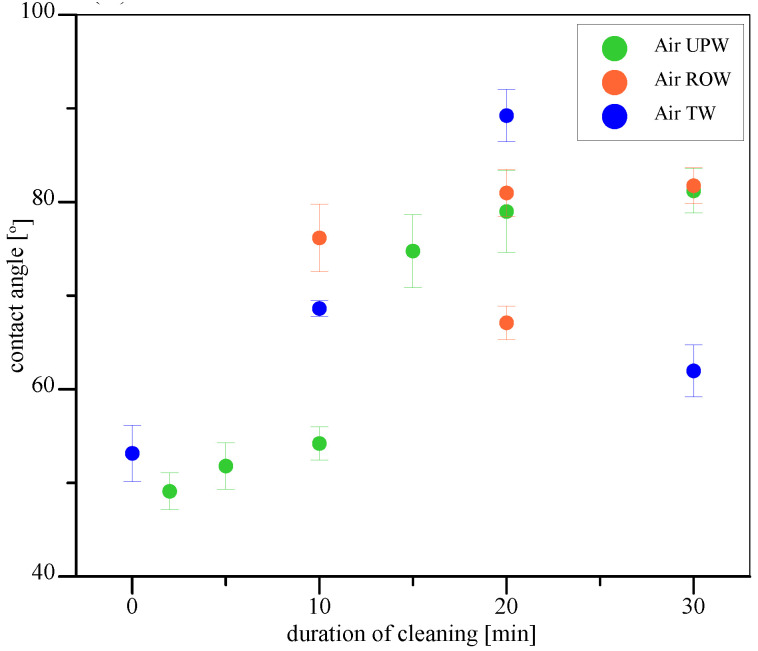
Contact angles of water on stainless steel surfaces contaminated with Pluronic L-121 before and during cleaning with air microbubbles in ultrapure water (green), reversed-osmosis water (red), and tap water (blue).

**Figure 2:**
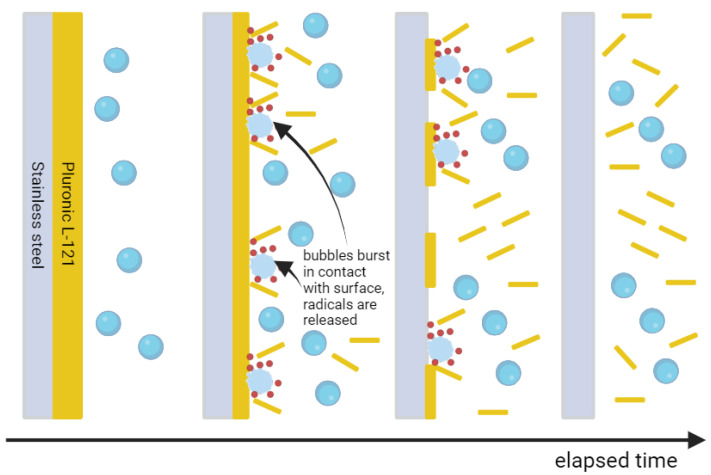
Stages of cleaning with air microbubbles in ultrapure water. From left to right: microbubble concentration increases; next, the bubbles impact the surface of contamination and burst, releasing the hydroxyl radicals (red dots); gradually, the thickness and continuity of the contamination layer are reduced; the bubbles stay in liquid and prevent recontamination.

The first measurements were performed for ultrapure water (UPW) and air microbubbles (MB-AIR). Six durations of cleaning were considered: 2, 5, 10, 15, 20, and 30 min. One can see from the red points in Figure 1 that the cleaning curve is following the sigmoidal path. This effect is connected to the different stages of cleaning visualized in Figure 2; we observe only a slight increase in the contact angle for the first 10 min of cleaning. We suspect that this is connected to the removal of the outer layers of contamination without significant disruption of the continuity of the coverage of the steel plate. This is backed by the fact that the standard deviation of results for each duration of cleaning between the 0th and 10th minute is diminutive, which shows the uniformity of the surface coverage by contaminant. Between the 10th and 15th minute, we see the drastic increase in contact angle, which hints that the continuous layer of contamination was broken and only a slight margin of the hydrophilic coating remains on the steel surface. For the next 15 min, the contact angle slowly increases up to the value obtained for the pure stainless-steel surface before contamination. It is also worth noting that the standard deviation of results after 30 min is much lower than for 15 or 20 min of cleaning, which also confirms that the surface is uniform and there is no residue of contamination. We suspect that the microbubbles impact the contaminated surface, and during impact, they burst. The energy of such a burst causes the hydrogen radical release (red dots in Figure 2), as reported in the literature [50,51,52].

**Figure 3:**
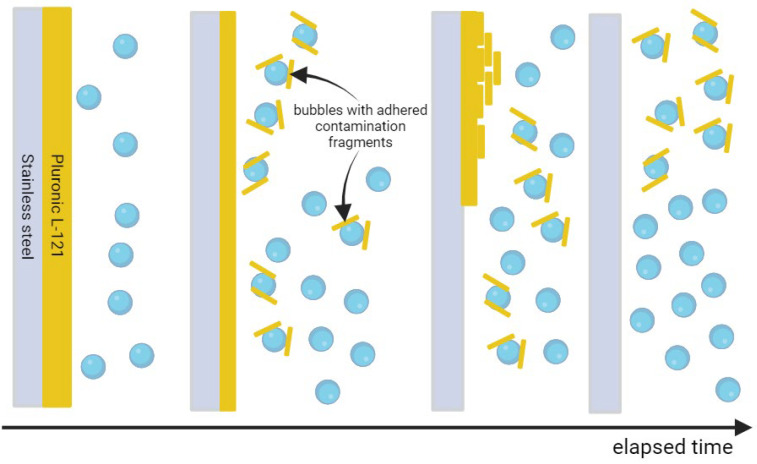
Stages of cleaning with air microbubbles using water after reversed osmosis. From left to right: microbubble concentration increases, microbubbles detach and float parts of the contamination, contamination is accumulated in higher places of the plate, and accumulated material is floated further to the surface of the liquid.

The time of 30 min was enough to clean the Pluronic L-121 contamination from stainless steel, i.e., the contact angle on the whole plate was homogeneous and close to the value obtained for pure stainless steel before contamination. Therefore, 30 min was set as the default duration of the cleaning process. Water after reversed osmosis (ROW) was taken next, with air microbubble dispersion used to clean off the contamination. What is visible as red points in Figure 1 is that even though the ROW water at first looks less pure than UPW, the increase in the contact angle to values pointing to the destruction of contamination layer continuity is visible sooner for ROW than in the case of UPW. One possible explanation for such a phenomenon is the presence of a higher number of ions in the ROW compared to UPW. Larger ion presence enhances the bubble stability as it allows for the easier formation of ion shells, which was shown by various researchers [46,53,54,55,56,57,58]. Therefore, the microbubbles that encounter the contamination on the surface can interact with it, squeeze into imperfections of the contamination layer, and remove the contaminant. Additionally, as the bubbles stabilized by the ions have higher longevity in the bulk of the liquid, their concentration should also be higher. Both these effects affect the cleaning potential of microbubble-enriched water. Additionally, one can note that, for 20 min of cleaning, two distinct values of contact angles were repeatably measured for respective spots on several investigated steel plates. The values were independent on the plate used, i.e., they were similar for the same spots on different plates. We suspect that during cleaning, the contamination accumulated in one part of the plate (lower values of contact angle) due to the flotation effect of nanobubbles, while other parts were cleaned completely. After an additional 10 min, the value of the contact angle was uniform on the whole surface of the investigated steel plates, and for each plate, the same value was obtained. The stages of cleaning, in this case, are visualized in Figure 3.

Results obtained for the tap water (TW; blue points in Figure 1) were at first the most surprising and puzzling. The values of contact angles between the 0th and 20th minute were increasing, which indicates a good cleaning rate, and the value for 20 min of cleaning far surpassed the contact angle for a clean stainless-steel plate. However, the next point, acquired after 30 min of cleaning, had an extremely low value, only slightly larger than the value acquired for the contaminated plate, which would indicate that no cleaning took place. Additional repetitions were carried out for this experiment, and they have shown the same result. We suspect that the initial change in hydrophobicity is connected to the formation of micropancakes or nanobubbles on the steel surface, which, when the critical concentration of bubbles is exceeded, are subjected to coalescence and further detachment from the surface due to buoyancy. According to this suspicion, this layer of gas is adsorbed onto the contamination and does not cause any breaks in the contaminant coverage of the steel surface.

**Figure 4:**
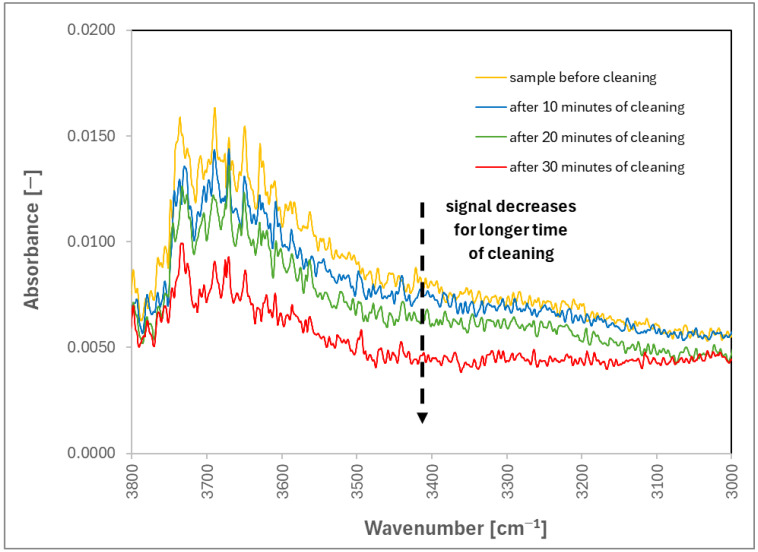
FTIR spectra for cleaning with tap water: sample before cleaning (yellow), sample after 10 min of cleaning (blue), sample after 20 min of cleaning (green), and sample after 30 min of cleaning (red).

**Figure 5:**
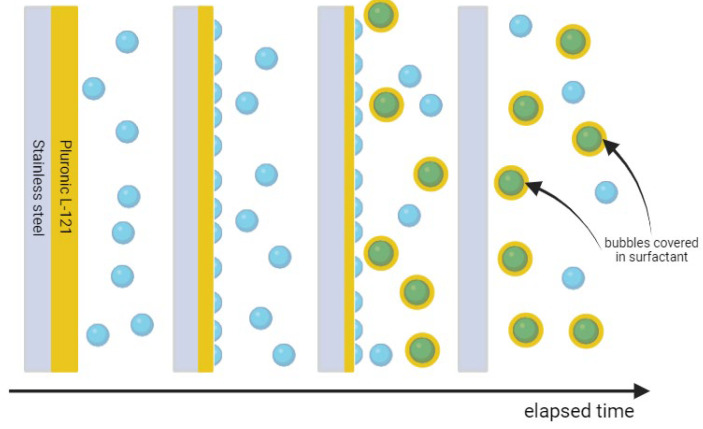
Stages of cleaning with air microbubbles using tap water. From left to right: microbubble concentration increases, microbubbles attach to the surface, bubbles rise in diameter and detach with part of the contamination adsorbed onto its surface, and accumulated material is floated further to the surface of the liquid.

To check whether cleaning occurs, the FTIR (Fourier Transform Infrared Spectroscopy) measurements were employed, which will be able to show the change in surface concentration of Pluronic L-121, which is the block-co-polymer of poly(ethylene oxide) (i.e., PEO) and poly(propylene oxide) (i.e., PPO), and therefore visible in FTIR spectrum. However, the FTIR measurements (Figure 4) showed that the amount of Pluronic L-121 was significantly reduced between the contaminated state and time points during cleaning. For example, the slope for frequencies 1100–1400 is less steep the longer the cleaning, indicating the reduction in PPO and PEO concentrations on the surface. For that reason, the suspicion that no cleaning takes place was abandoned. However, that still left us with the existence of this peculiar maximum for tap water in Figure 1. Interestingly, we have realized that the adsorption of gas bubbles on the surface of contamination and their subsequent coalescence and detachment are not contradictory to the reduction in contaminant concentration on the steel surface. We suspect that during coalescence and detachment, the newly formed bubble is also carrying part of the contamination with it, as visualized in Figure 5.

**Figure 6:**
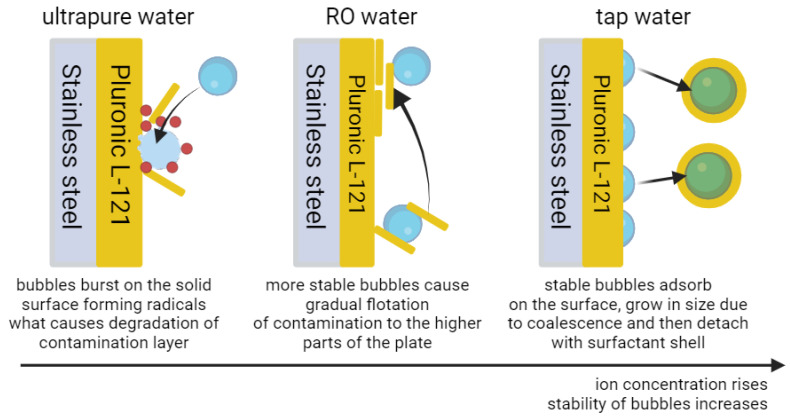
Comparison of most dominant interactions between air microbubbles and contamination for waters of different purities.

One may ask why no such effect was visible for both UPW and ROW. In our opinion, the effect is connected to microbubble stability in waters of different ion concentrations. In UPW, where the lowest concentration of ions is, the stability of microbubbles is also the lowest. As the bubbles are not stable, they burst easily, and a long time is needed to accumulate the high concentration of microbubbles. However, when such concentration is reached, bubbles frequently hit the contaminated surface, where they burst, exerting a high energy spike locally, which allows for radical formation. Such radicals, mostly hydroxyl radicals from water breakage, can react with the contaminant and degrade it significantly. That explains the long windup of cleaning (hardships with reaching high bubble concentration) and uniform cleaning of the whole surface (homogeneous dispersion of bubbles hitting the whole surface equally). For ROW, which has a higher ion concentration, the bubbles are more stable than for UPW, and therefore, the time of bubble saturation of dispersion is shorter. However, due to higher stability, such bubbles have a lower chance of bursting, which causes non-uniform cleaning. Also, as fewer hydroxyl radicals are generated, the contamination is not degraded as much as in the case of UPW; whole layers of contamination may detach from one place on the surface and then accumulate somewhere else. The place of accumulation is the same for every plate, which is precisely in the upper parts and indicates that the effects of the liquid hydrodynamics during microbubble formation have nearly no effect on the accumulation process; the transfer of contamination is probably flotation-based. Lastly, for TW, where the concentration of ions is the highest of all three waters, the bubbles also tend to be the most stable. For that reason, instead of bursting, the bubble adsorption, followed by coalescence and detachment (with part of the contamination adhered to the bubble surface), occurs. As contamination is floated on the large bubble surface, it cannot be accumulated in another place on the plate. These effects are visualized in Figure 6. One important note is that we suspect that all these mechanisms take place for each of the water purities (i.e., mostly ion concentration); however, the stability of bubbles determines which one is more dominant. Of course, for bubbles to be able to float the contamination, such contamination must already be in the bulk; therefore, some detachment due to bursting may have taken place. On the other hand, for the surface to be resistant to recontamination, the bubbles may form a protective layer by adhesion to the steel surface, analogously to the concept proposed by Zhu et al. (2016). Therefore, all these mechanisms are complementary and support one another. It is also worth considering that the nanobubbles in pH close to neutral are negatively charged [46,47,56,59] and, therefore, will attract positively charged particles or molecules while repelling those with negative charge exposed on the surface, what may affect cleaning process [60].

It is worth noting that for millimeter-sized bubbles, there is a thorough study of the effect of salt concentration on coalescence (see, e.g., review article by Firouzi et al. [61]). For such bubbles, there are defined concentrations of specific ions that cause the bubble stabilization and prevent its coalescence with surrounding bubbles [62,63]. However, all the studies in such matter were carried out for millimeter-sized bubbles. For smaller bubbles, some results prove that nanobubbles are destabilized by the excess presence of ions even in low concentrations [47,64]. Based on this fact, and as TW has the highest concentration of ions, it backs up our hypothesis about their coalescence and detachment from the solid surface.

**Figure 7:**
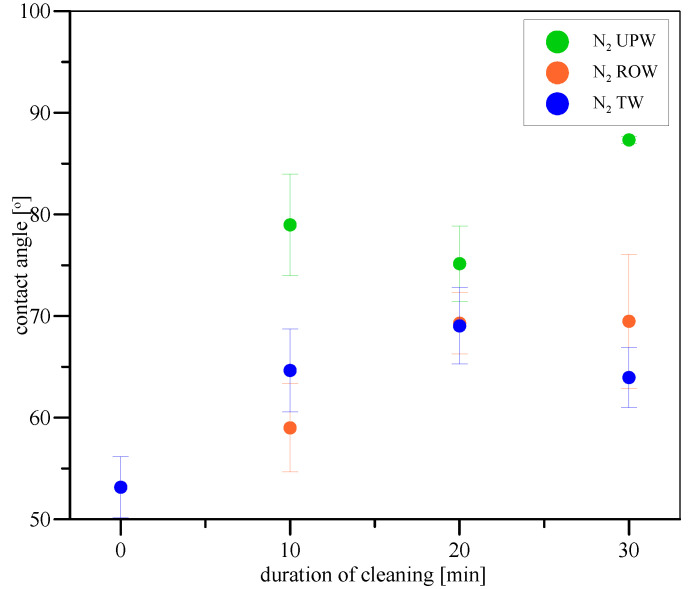
Contact angles of water on stainless steel surfaces contaminated with Pluronic L-121 before and during cleaning with nitrogen microbubbles in ultrapure water (green), reversed-osmosis water (red), and tap water (blue).

Interestingly, when the gas in microbubbles was changed from air to nitrogen, the effects of cleaning were extremely different. As visible in Figure 7, pure nitrogen in microbubbles has managed to clean the Pluronic L-121 contamination only using UPW during 30 min of cleaning, while ROW and TW were not able to pierce through the contamination layer and disrupt it during 30 min of cleaning. Therefore, one may suspect that lack of oxygen has affected the cleaning efficiency of microdispersion. This also provides additional facts backing our suspicion that radicals are formed during microbubble breakage, which is easier to generate when the environment is oxygen-rich. Because of the smaller chance of radical formation and overall lower reactivity of nitrogen compared to air, the mechanical part of cleaning is probably more effective. Still, the UPW allows for the easiest radical (originating from nitrogen) formation from all three waters, even when dispersion lacks oxygen, which allows it to clean the surface most efficiently. Additionally, one extremely interesting effect occurred after 30 min of cleaning with nitrogen-microbubble-enriched UPW. The contact angle became higher than for pure stainless steel. This shows that the surface became more hydrophobic than the original, and it points to changes in the surface microstructure. Such changes, as suspected in the case of TW and air microbubbles, may be due to the effect of microbubble adhesion on the surface of the stainless steel. As fewer radicals are formed, the chain reaction of microbubble bursting due to the presence of radicals in the bulk of the liquid is less common; therefore, adhesion is more probable. Therefore, the mechanisms for nitrogen microbubbles tend to be skewed toward those encountered for air microbubbles in tap water, i.e., when the stability of the microbubbles was the highest due to the shielding effects of ion shell.

**Figure 8:**
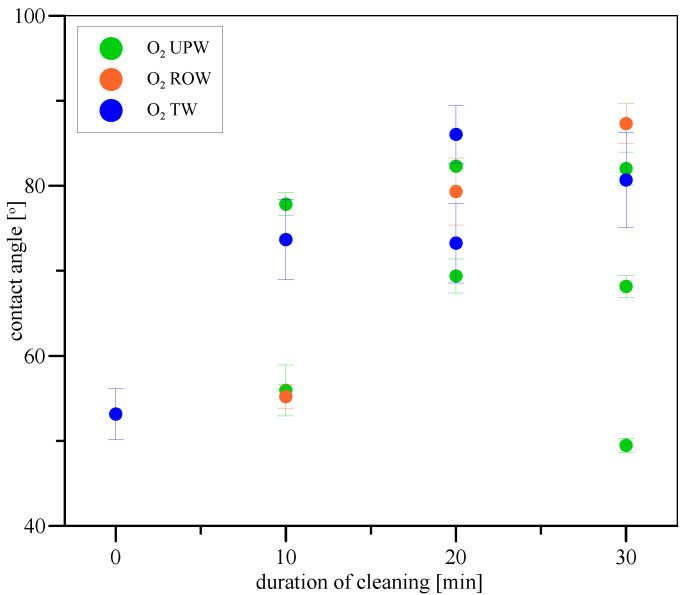
Contact angles of water on stainless steel surfaces contaminated with Pluronic L-121 before and during cleaning with oxygen microbubbles in ultrapure water (green), reversed-osmosis water (red), and tap water (blue).

In the case of oxygen microbubbles, the results were not surprising as they were the direct extrapolation of the hypothetical description of the phenomena encountered for air and nitrogen nanobubbles. For UPW and oxygen microbubbles (Figure 8, green line), the cleaning of the plates was extremely non-uniform. For each duration of cleaning, we could find at least two distinct values of contact angle, which were measured repeatably for different plates. As stated above, results have not surprised us, as similar results, even though less apparent, were obtained for ROW and air microbubbles, while nitrogen microbubbles have not caused the formation of any non-uniformity on the cleaned steel surface. We see this as the function of increasing the oxygen concentration, which changes the ratio of influence of different mechanisms visualized in Figure 7. For pure nitrogen in microbubbles, we observe high uniformity of cleaning but with lower efficiency. Air microbubbles in UPW allow for deep cleaning, which is not thorough across the plate surface, while oxygen microbubbles also allow for deep cleaning, but the level of non-uniformity is even higher than for air microbubbles. We suspect that due to high reactiveness, oxygen microbubbles allow for chemical degradation of the contamination, but for the same reason, no mechanical and thorough cleaning takes place, as bubbles have nearly no chance for stable adhesion to the contamination surface. The chemical reaction occurs directly at the place of microbubble impact with the plate, which, based on the placement of plates in the vessel, took place in the lower half of the vertically oriented plates.

What is especially interesting is that a similar effect was not replicated when the water was changed to ROW (red points in Figure 8). These results were extremely similar to those obtained for UPW for air microbubbles. That shows that the small addition of ions in the solution significantly lowers the cleaning power of oxygen nanobubbles in the first minutes of the cleaning when the microbubble number concentration is still low. That would mean that the reactiveness of the oxygen microbubbles was hindered. However, when the critical number is exceeded, the cleaning is rapid. Contrary to the air microbubbles, we observe a higher hydrophobicity than the original hydrophobicity of stainless steel for 30 min of cleaning, which indicates a change in the microstructure of the surface. The results obtained are also extremely uniform, both within one plate and between them, which shows the high repeatability of the cleaning process.

In the case of oxygen microbubbles in TW (blue points in Figure 8), the results are also similar to those obtained for air microbubbles. The initial, rapid increase in the value of the contact angle is visible, up to the value exceeding the contact angle for pure stainless steel, which is followed by the decrease between the 20th and 30th minute. These results once again point toward the suspicion that the bubbles form the micropancakes or surface microbubbles onto the surface, which then reside there until the coalescence occurs and they are detached from the surface. It is also possible for oxygen microbubbles since the reactivity of oxygen is hindered by the presence of an ion shell, which surrounds the bubbles and prevents them from bursting or coalescing in bulk as a response to impact with a solid surface or other bubbles. That leads us to the conclusion that the interactions between oxygen microbubbles and contamination are closer to the phenomena encountered in air microbubbles in water than in the case of nitrogen nanobubbles.

## 3. Materials and Methods

### 3.1. Preparation of Contaminant Solutions

Pluronic L-121 (Merck, Darmstadt, Germany) solution ($500\frac{\mu L}{L}$) was prepared by pouring 500 μL of Pluronic L-121 into the 1000 mL volumetric flask and filling the flask to marked volume. The mixture was stirred using a magnetic stirrer.

### 3.2. Contaminated-Stainless-Steel Preparation

Stainless steel plates (100 × 20 × 5 mm) were rinsed with water and ethanol to get rid of potential impurities and left to dry. Next, the plates were hung onto the horizontal rods over the contaminant solution using clamps. Plates were lowered into the contaminant solution for 15 min for coating. The solution was gently stirred using a magnetic stirrer for the whole duration of the coating. Then, plates were hung over paper towels and left to dry.

### 3.3. Water Preparation

Water of three different purities was used: tap water from the laboratory (conductivity of $0.8\frac{mS}{cm}$) and two purified waters obtained using the modified Deioniser DL3-100 (Polwater, Kraków, Poland)—water after reversed osmosis (conductivity of about $6.0\frac{\mu S}{cm}$) and ultrapure water (conductivity $<0.05\frac{\mu S}{cm}$). All water samples were used immediately, without any storage.

### 3.4. Cleaning Procedure

**Figure 9:**
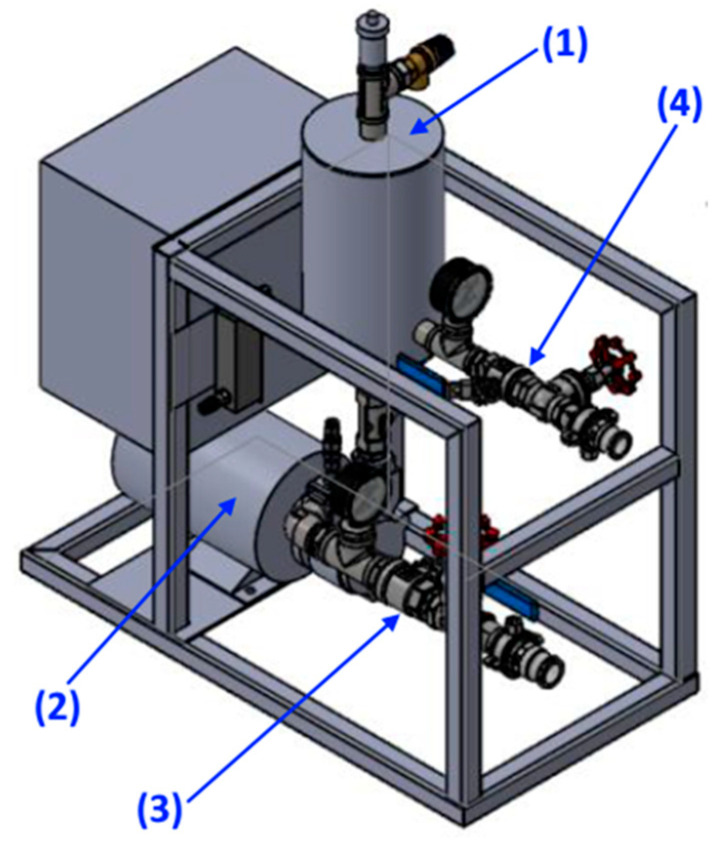
Scheme of the setup with KTM 25 system. The setup is composed of the KTM 25 microbubble generator (1) with non-return gas valve, tank for gas bubble segregation (2) with vent valve and two manometers, and two sets of regulating and shut-off valves on the suction (3) and discharge (4) pipes. During the microbubble generation, water is circulating between the setup and storage tank (not shown in the image). The setup was manufactured by Fine Bubble Technologies (Piaseczno, Poland).

For cleaning contaminated samples, the system with KTM 25 (Nikuni, Kanagawa, Japan), which can produce microbubbles, was used (Figure 9). The tank was filled with $40\;dm^{3}$ of water of a given quality (either tap water, TW, water after reversed osmosis, ROW, or ultrapure water, UPW). Water was circulated through the pump, with the gas delivered on the suction side. A two-phase mixture is circulated between the tank and the pump for the whole duration of cleaning. After about 10 s from the start of the pump engine, the so-called “white water” was visible. According to the manufacturer, the diameter of generated bubbles is in range between 8 and 15 μm. Depending on the experiment, either atmospheric air, nitrogen, or oxygen were supplied to the gas inlet of the pump. The flow rate of the gas-liquid mixture was set to $0.5\frac{dm^{3}}{s}$. Three contaminated steel plates were hung onto the holder using clamps and immersed in the liquid. To minimize the effect of the shear stress on the cleaning process, the plates were immersed far from the inlet of the tank, i.e., outside of the stream coming from the pump. The cleaning process was carried out for a given time, and after that, designated plates were taken out of the liquid and left to dry in the atmospheric conditions.

### 3.5. Sample Range

The combinations of water of 3 different purities and 3 different gases supplied to the microbubble generator were investigated for the same operating conditions. Therefore, 9 sets of investigated samples were prepared.

Apart from the investigated samples, the reference samples were also prepared. The first kind of reference was the samples of stainless steel without contamination, serving as the aim of purification. The second reference was the samples of contaminated stainless steel without cleaning, being the starting point of the cleaning. Lastly, to check whether the presence of microbubbles affects the cleaning, the samples immersed for a given time in waters of given purity but without microbubble flow were prepared.

### 3.6. Measurements

The contact angles of water onto the samples were measured using goniometer DSA100 (Kruss, Hamburg, Germany), according to the following procedure: 5 μL droplet of ultrapure water was set onto the surface of the steel, and after 2 s (to allow the droplet to still itself), the images of the droplet were taken for 1 s with frequency of 10 fps. From each frame, the right and left angles were measured and then averaged to obtain the median angle. As the droplets were mostly symmetrical, the contact angle values on the left and right sides were extremely similar. Multiple droplets were analyzed in several places on the same plate to obtain information about the homogeneity of the surface. At least three technical replications of each experiment were performed (i.e., different plates were analyzed), and for each one, the corresponding places on the plates were analyzed.

Fourier Transform Infrared Spectroscopy with Attenuated Total Reflection (FTIR-ATR) measurements were carried out using Nicolet 6700 (ThermoScientific, Waltham, MA, USA).

## 4. Conclusions

Three different gases—oxygen, air, and nitrogen—were enclosed in microbubbles, and waters of three different purities were used to check the influence of both the content of the gas phase and the ion concentration of the liquid phase (assumed to be proportional to the conductivity).

Results showed that an increase in ion concentration in the liquid surrounding the microbubbles leads to a change in the dynamics and mechanisms of cleaning the stainless-steel plates. Based on the results obtained for air microbubbles, we postulated that three different mechanisms occur, but the ionic strength of the liquid phase determines which one dominates over the other two. For the highest purity water (ultrapure water, conductivity $<0.05\frac{\mu S}{cm}$), the microbubbles easily burst during impact with the surface, releasing hydroxyl radicals, which allows for the chemical decomposition of contamination in addition to the mechanical detachment of contaminant fragments. For water of intermediate purity (water after reversed osmosis; conductivity $\sim 6.0\frac{\mu S}{cm}$), the stability of bubbles is higher; therefore, they can detach small parts of the contamination during impact and float them to the higher parts of the plate. The proof is the gradual change in contact angle of the lower parts of the plates, while, for the higher parts, it remained nearly the same. Lastly, for the water of the lowest purity (tap water, conductivity $800\frac{\mu S}{cm}$), the stability of bubbles is so high that they are observed on the surface of the plate even after it is removed from dispersion. As the cleaning was also uniform, we state that the microbubbles adhere to the surface of contamination and, after coalescence, detach with the fragments of contamination adhering to their surface. Unfortunately, this mechanism is ineffective compared to previous ones as air microbubbles in tap water were unable to clean off the contamination.

As for the change in oxygen content in microbubbles when the nitrogen microbubbles were employed, the cleaning effectiveness was drastically reduced. Only the ultrapure water containing nitrogen microbubbles was able to clean off the contamination during 30 min of cleaning. That leads to the conclusion that the lower oxygenation potential of nitrogen microbubbles leads to a lower number of hydroxyl radicals being released and, as a consequence, lower effectiveness of cleaning. On the other hand, when the oxygen microbubbles were used, the effectiveness of cleaning was the lowest for ultrapure water. We postulate that because of the extremely high reactivity of oxygen microbubbles, they cannot interact mechanically with the contaminant, and therefore, only chemical interactions occur.

Therefore, in this paper, the effect of both the content of liquid and gas phases was investigated, and the clear effects of both were shown. In our opinion, the next step of the studies of this matter will involve gases of different structures and reactiveness (such as carbon dioxide) as well as investigate the effect of the addition of medically approved detergents. In the work presented here, the attempt was made to maintain only the effect of the microdispersion on the cleanable surface without the flow hydrodynamics affecting the flow in a significant manner. The separate and very broad issue will be the combination of these results with a dynamic cleaning effect, i.e., the addition of a mechanical removal of contamination. In the case of microdispersion, this can be carried out by simply directing the flow onto the surface to perpendicularly impact it with the water jet from the generator. We hope that our results will become an interesting step in the intensification of the cleaning process with nanobubbles and microbubbles. We think that the possibility of controlling the dominance of specific mechanisms in cleaning can be potentially used in cleaning processes in industry.

## Figures and Tables

**Table 1:** The contact angle of ultrapure water in air measured on stainless steel samples.

Sample No.	Water Used	Microbubbles Used	Time of Cleaning [min]	Median Contact Angle [°]	Notes
1	N/A	N/A	N/A	82.01±1.39	Purified stainless steel without contamination
2	N/A	N/A	N/A	53.15±3.01	Samples with contamination before cleaning
3	UPW	N/A	30	50.28±1.86	Contaminated samples immersed in given water without microbubble generation
4	ROW	N/A	30	54.83±1.37	
5	TW	N/A	30	58.75±3.00	
6	UPW	MB-AIR	2	49.10±1.96	
7	UPW	MB-AIR	5	51.79±2.48	
8	UPW	MB-AIR	10	54.21±1.76	
9	UPW	MB-AIR	15	74.76±3.92	
10	UPW	MB-AIR	20	78.98±4.36	
11	UPW	MB-AIR	30	81.20±2.39	
12	ROW	MB-AIR	10	76.17±3.62	
13	ROW	MB-AIR	20	80.97±2.54 67.10±1.79	Two distinct contact angle values were measured for different parts of the investigated plates
14	ROW	MB-AIR	30	81.74±1.90	
15	TW	MB-AIR	10	68.62±0.86	
16	TW	MB-AIR	20	89.23±2.80	
17	TW	MB-AIR	30	61.96±2.78	
18	UPW	$MB-N_{2}$	10	78.97±5.00	
19	UPW	$MB-N_{2}$	20	75.14±3.71	
20	UPW	$MB-N_{2}$	30	87.32±0.33	
21	ROW	$MB-N_{2}$	10	58.99±4.34	
22	ROW	$MB-N_{2}$	20	69.29±3,02	
23	ROW	$MB-N_{2}$	30	69.48±6,58	
24	TW	$MB-N_{2}$	10	64.64±4.09	
25	TW	$MB-N_{2}$	20	69.03±3.75	
26	TW	$MB-N_{2}$	30	63.94±2.97	
27	UPW	${MB-O}_{2}$	10	77.82±1.35 55.94±3.00	Two distinct contact angle values were measured for different parts of the investigated plates
28	UPW	${MB-O}_{2}$	20	82.29±0.24 69.38±2.01	Two distinct contact angle values were measured for different parts of the investigated plates
29	UPW	${MB-O}_{2}$	30	82.02±1.95 68.17±1.27 49.46±0.84	Three distinct contact angle values were measured for different parts of the investigated plates
30	ROW	${MB-O}_{2}$	10	55.19±1.40	
31	ROW	${MB-O}_{2}$	20	79.32±3.95	
32	ROW	${MB-O}_{2}$	30	87.31±2.35	
33	TW	${MB-O}_{2}$	10	73.66±4.70	
34	TW	${MB-O}_{2}$	20	86.04±3.39 73.25±4.68	Two distinct contact angle values were measured for different parts of the investigated plates
35	TW	${MB-O}_{2}$	30	80.48±6.12	

UPW—ultrapure water, ROW—water after reversed osmosis, TW—tap water, MB-AIR—atmospheric air microbubbles, $MB-N_{2}$—nitrogen microbubbles, ${MB-O}_{2}$—oxygen microbubbles.

## Data Availability

The original contributions presented in this study are included in the article. Further inquiries can be directed to the corresponding author.
